# Autosomal recessive primary microcephaly type 2 associated with a novel *WDR62* splicing variant that disrupts the expression of the functional transcript

**DOI:** 10.3389/fneur.2024.1341864

**Published:** 2024-03-21

**Authors:** Haizhu Chen, Ying Zheng, Hua Wu, Naiqing Cai, Guorong Xu, Yi Lin, Jin-Jing Li

**Affiliations:** ^1^Department of Neurology, Institute of Neurology of First Affiliated Hospital, Institute of Neuroscience, and Fujian Key Laboratory of Molecular Neurology, Fujian Medical University, Fuzhou, China; ^2^Department of Neurology and Institute of Neurology of First Affiliated Hospital, National Regional Medical Center, Binhai Campus of the First Affiliated Hospital, Fujian Medical University, Fuzhou, China

**Keywords:** microcephaly, autosomal recessive primary microcephaly type 2, *WDR62* mutation, epilepsy, consanguineous marriage, brain malformation

## Abstract

**Background:**

Autosomal recessive primary microcephaly (MCPH) is a rare neurodevelopmental disorder characterized primarily by congenital microcephaly and intellectual disability but without extra-central nervous system malformations. This investigation aimed to elucidate the genetic underpinnings of microcephaly in a patient from a Chinese consanguineous family.

**Methods:**

A comprehensive clinical assessment, including brain magnetic resonance imaging (MRI), electroencephalogram (EEG), and genetic analyses, was conducted to evaluate the patient’s condition. Whole-exome sequencing (WES) was employed to identify the causative gene, followed by Sanger sequencing, to confirm the mutation and its segregation within the family. Reverse transcript polymerase chain reaction (RT-PCR) was utilized to detect changes in splicing. Western blot was employed to reveal the difference of protein expression level between the wild-type and mutant WDR62 in vitro.

**Results:**

The patient exhibited classic MCPH symptoms, including microcephaly, recurrent epilepsy, delayed psychomotor development, and intellectual disability. Additionally, asymmetrical limb length was noted as a prominent feature. MRI findings indicated reduced brain volume with cortical malformations, while EEG demonstrated heightened sharp wave activity. A molecular analysis uncovered a novel homozygous variant c.4154–6 C > G in the *WDR62* intron, and a functional analysis confirmed the pathogenicity of this mutation, resulting in the formation of an abnormal transcript with premature termination codons.

**Conclusion:**

This study enhances our understanding of the genetic heterogeneity associated with MCPH and highlights the pivotal role of genetic testing in the diagnosing and managing of rare neurodevelopmental disorders. Furthermore, it highlights the potential of emerging genetic therapies in treating conditions such as MCPH2.

## Introduction

Autosomal recessive primary microcephaly (microcephaly primary hereditary, MCPH) is a rare neurodevelopmental disorder characterized by congenital microcephaly and intellectual disability but without extra-central nervous system malformations (1). The prevalence of MCPH varies by geographic region or marriage customs, typically occurring in 1:30,000 to 1:250,000 live births (2). It ranges from 1 in 2,000,000 in Scotland, 1 in 250,000 in Holland, and 1 in 30,000 in Japan to 1 in 10,000 in areas where consanguineous marriages are common (3–7). MCPH exhibits high genetic and phenotypic heterogeneity. To date, 30 pathogenic genes corresponding to MCPH1-MCPH30 have been reported, with the *ASPM* gene being the most common pathogenic gene, accounting for 50%, and the *WDR62* (WD40-repeat protein 62) gene, corresponding to MCPH2, accounting for 10% (8, 9).

MCPH2, the second most frequent type of MCPH, is characterized by severe motor handicap, epilepsy, and intellectual disability and is associated with a poor prognosis (10–12). Brain magnetic resonance imaging (MRI) in MCPH2 patients typically reveals a reduction in brain volume and cortical malformations, including neuronal heterotopia, pachygyria, schizencephaly, and microlissencephaly (11, 13–18).

The *WDR62* gene contains 32 exons and encodes a 1,523-residue protein associated with the microtubule minus-end mitotic spindle pole, which is highly expressed in the forebrain during neurogenesis, particularly in the ventricular and subventicular zones, and plays a vital role in neuronal cell proliferation and migration (19). As *WDR62* was identified as a causative gene for MCPH2 (11, 12), over 50 pathogenic variants have been reported, including missense mutations, duplications, insertions, deletions, stop-gain mutations, and splice-site substitutions. The application of next-generation sequencing (NGS) has improved the diagnosis rate of primary microcephaly and facilitated the identification of disease subtypes, significantly reducing time and labor costs.

In this study, we describe a patient from a consanguineous family harboring a novel splicing site mutation in c.4154-6C > G of *WDR62* detected by whole-exome sequencing (WES). The patient was diagnosed with autosomal recessive primary microcephaly, and our aim was to evaluate the clinical features, brain MRI, electroencephalogram (EEG), and genetics of the proband.

## Materials and methods

### Subjects and sample collection

The proband, accompanied by his parents and siblings, was admitted to our Neurology Department at the First Affiliated Hospital of Fujian Medical University with the chief complaint of recurrent epileptiform seizure. Brain magnetic resonance imaging (MRI) scan, electroencephalogram (EEG), electrocardiogram (ECG), ambulatory blood pressure monitoring, and echocardiography were utilized to assist in the diagnosis of the disease. Diagnosis was confirmed by two experienced neurologists. Peripheral blood samples were collected from all family members, and genomic DNA was extracted using the QIAamp DNA Blood Mini Kit (QIAGEN, CA, USA) according to the manufacturer’s instructions. This study was approved by the ethics committee for Medical Research of the First Affiliated Hospital of Fujian Medical University (FYYY2006-01-1901), and written informed consent was obtained from all participants.

### Whole-exome sequencing

Whole-exome sequencing (WES) was performed on the proband using Agilent SureSelect V6 capture kits (Aglient Technologies Co., Ltd) on the Illumina HiSeq 2,500 platform. Raw data were converted to FASTQ files, and low-quality reads were eliminated. Clean FASTQ formatted sequences were aligned to the human reference genome using BWA-MEM. Variant calling was conducted using GATK haplotype caller, and annotation was performed using ANNOVAR. Only variants fufilling a recessive inheritance pattern with a frequency of less than 1% in the gnomAD population, combined with the preliminary clinical diagnosis, were screened.

### Sanger sequencing

To confirm the variants identified through a WES analysis, specific primers were designed for amplification of the *WDR62* mutations. The targeted regions were polymerase chain reaction (PCR) amplified in a C1000 Touch Thermal Cycler (Bio-Rad). Following purification, the amplified products were electrophoresed on an ABI 3730xl automated DNA analyzer (PE Applied Biosystems, Foster City, CA) according to standard protocols. The sequencing results were aligned with reference genomes downloaded from Ensembl.^1^

### RNA isolation and reverse transcription (RT)-PCR

RNA extraction from blood samples of the proband and the negative control was performed using TRIzol reagent (Invitrogen, CA, USA) following the manufacturer’s recommendations. For cDNA synthesis, we used 1 μg of each RNA sample per 20 μL reverse transcription reaction using a R333 HiScript® III All-in-one RT SuperMix (Vazyme Biotech Co, Ltd). The forward primer 5’-CTGAGACTGACCCTGTCAAGTGCCT-3′ and the reverse primer 5’-ACAGGAAGGTGGAGACCAGCTCAGT-3′ were designed to amplify the target region. The products were then used for agarose gel electrophoresis imaging to analyze the splicing alterations of the pre-mRNA affected by the splicing site variation.

### Plasmid construction and Western blot

The pCAG-Flag plasmid was linearized with BamHI and XhoI restriction endonuclease (Thermo Fisher Scientific), and primers were designed to amplify the target fragment. Plasmids were constructed using the NEBuilder HiFi DNA Assembly Master Mix (New England Biolabs) according to the standard manufacturer’s protocols. Two plasmids were constructed: One contained Flag-tagged *WDR62* cDNA with wild-type intron 30, and the other contained Flag-tagged *WDR62* cDNA with intron 30 containing the c.4154-6C > G variant. HEK293T cells were transfected with 2 μg of plasmids using Lipofectamine 3,000 (Thermo Fisher Scientific). After 48 h, the transfected cells were harvested. Protein extraction and Western blotting analysis were conducted using standard protocols, and four biological replicates were collected for statistical analysis (*t*-test). Differences were considered statistically significant when *p*-values were less than 0.05 (*), 0.01 (**), 0.001 (***), or 0.0001 (****). “ns” indicates not significant.

## Results

### Clinical description

**Figure 1:**
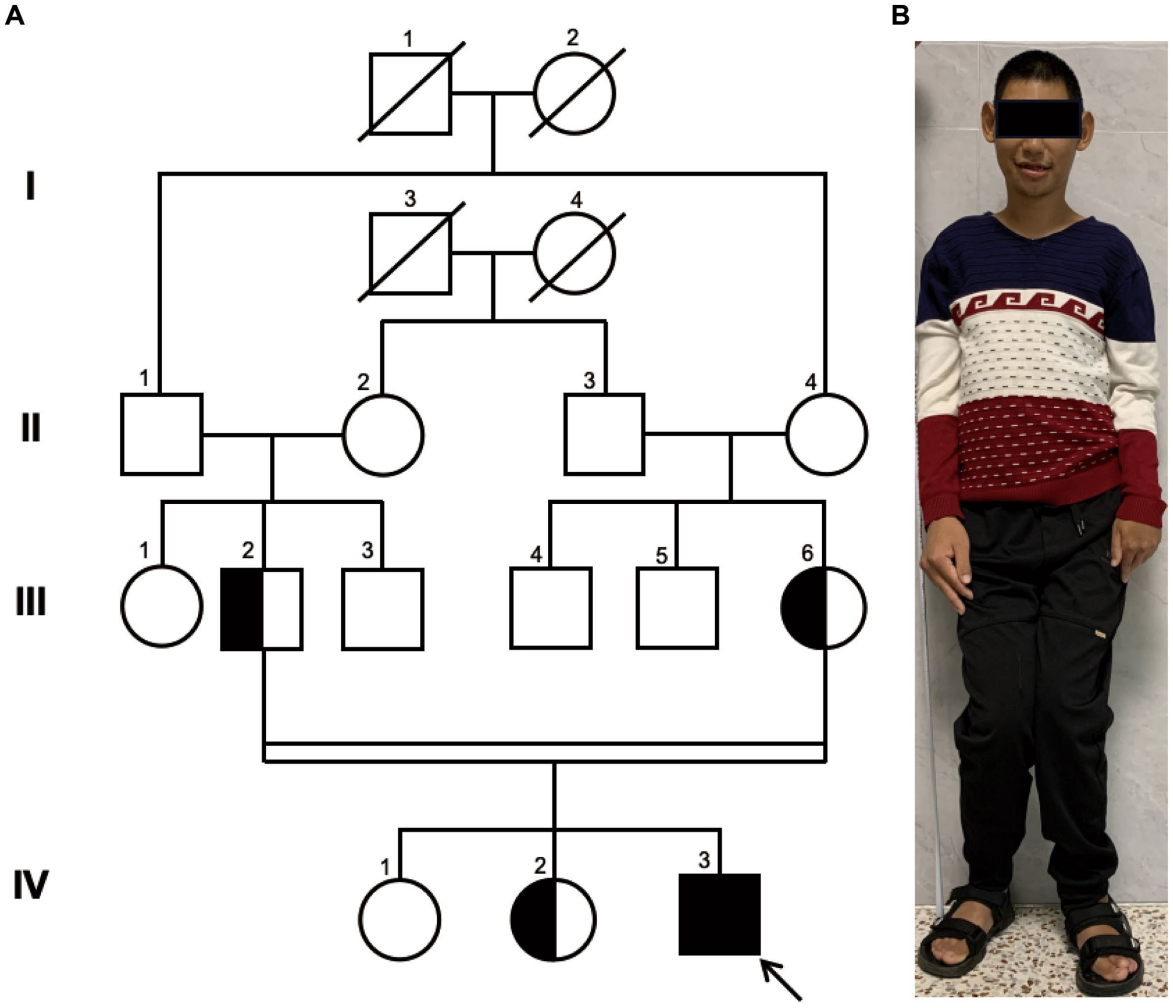
Family Pedigree and Photograph of Proband. **(A)** pedigree illustrating that the parents of the proband (III-2 and III-6) are first cousins, which represents a consanguineous marriage. The parents and the older sister are healthy carriers, while the eldest sister exhibits a normal genome in this site. Circles represent females, squares represent males, half-filled symbols indicate carriers, fully filled symbols indicate the affected individual, and the arrow points to the proband. **(B)** The full-body photograph of the proband showing a short stature and limbs of unequal length.

The proband, a 25-year-old male from a family of consanguineous marriage, exhibited phenotypical and behavioral characteristics of primary microcephaly, recurrent epilepsy, intellectual disability, limping gait, single-word speech, and an inability to walk in a straight line. The pedigree of the family is shown in Figure 1A. At the age of 1 month, he experienced hyperpyrexia lasting for 1 week. Since the age of 5 months, he had recurrent seizures occurring approximately once a month, which could be triggered by stimuli such as sound, temperature changes, falling, and limb touch. At 1 year of age, radiological imaging revealed dysplasia of the left femoral head. The patient was unable to crawl during infancy and began sitting independently at 3 years and moved his body with support from a bench. He achieved independent walking at 6 years of age and learned to use chopsticks at 8 years of age. Due to delayed psychomotor development, he did not receive formal education and never attended school. Seven years ago, he was admitted to a local hospital and started antiepileptic therapy with valproate acid and lamotrigine, which partially controlled his symptoms, although intermittent attacks persisted. At the age of 25 years, clinical evaluation revealed the patient’s stature was short (154 cm), with measurements indicating left upper limb length of 71.3 cm, right upper limb length of 76 cm, left lower limb length of 81 cm, right lower limb length of 84 cm, and head circumference of 50.5 cm (Figure 1B).

**Figure 2:**
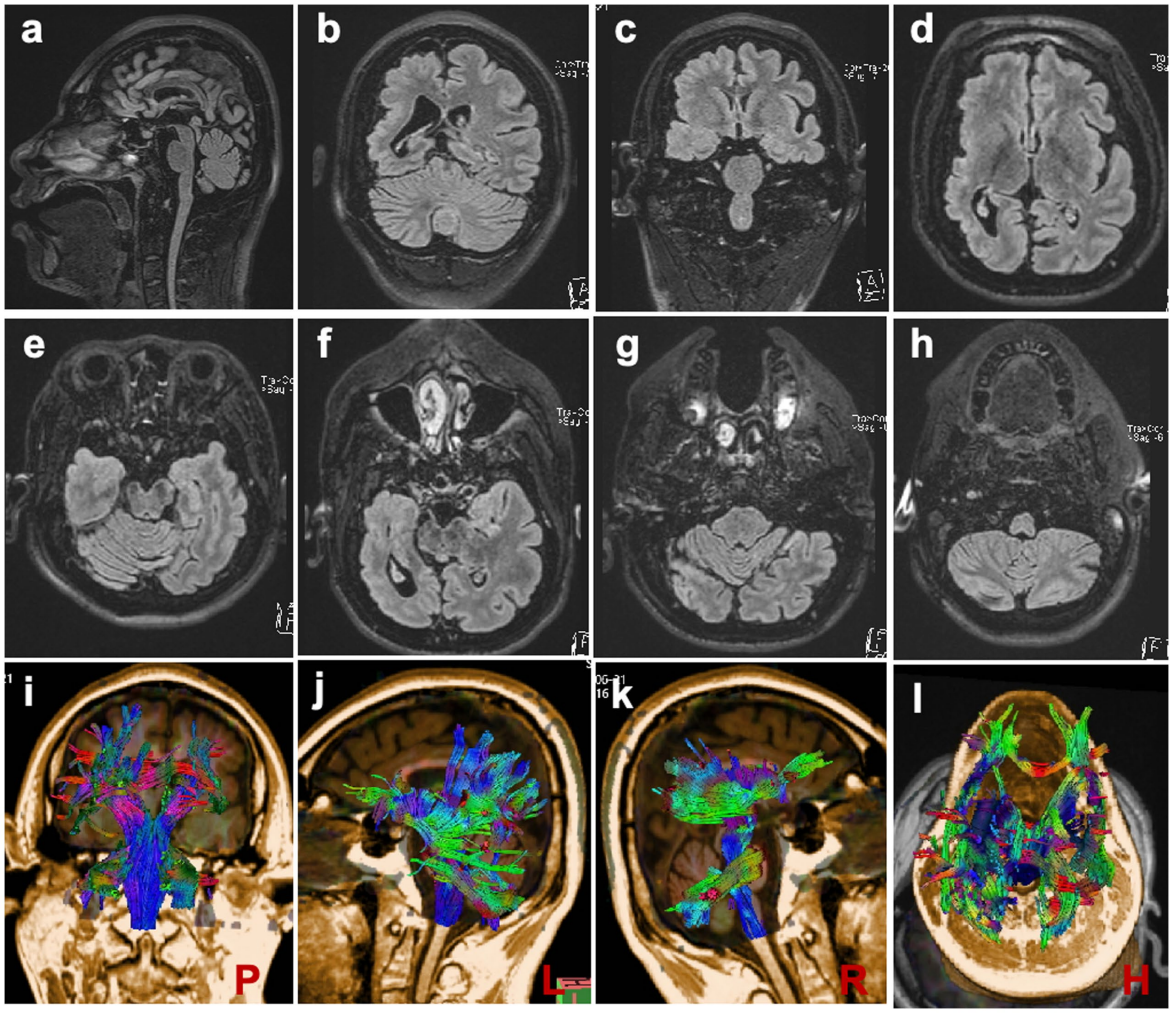
Brain MRI Scans **(A)** Scans demonstrating reduced brain volume and thinning of the corpus callosum. **(B–D, F)** Scans depicting widened sulci, reduced gyri, and dysplasia of the right cerebral hemisphere with bilaterally unequal ventricles. **(E–H)** Scans showing slight atrophy of the brain stem and cerebellum. **(I–L)** DTI series are presented at posterior, left, right, and horizontal angles, respectively.

**Figure 3:**
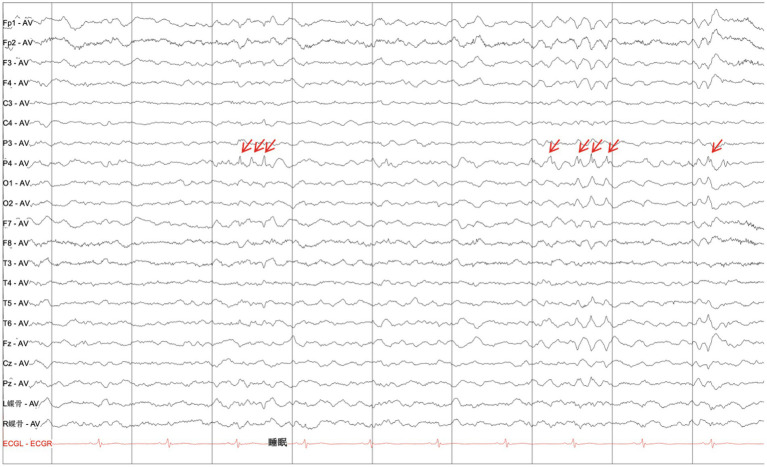
Electroencephalogram. EEG displaying a slight increase in the medium amplitude of the sharp waves and sharp-slow complex waves in the right parietal region during sleep (as indicated by red arrows).

MRI scans were performed on the proband to evaluate brain structures, revealing an obvious microcephaly with widened sulci and reduced gyri, thinning of the corpus callosum, particularly in the splenium, dysplasia of the right cerebral hemisphere with bilateral ventricles of unequal size, diffusely thickened cortex, loss of gray–white junction, and partial pachygyria. Schizencephaly was observed in the left parietal lobe, along with asymmetric atrophy of the mesencephalon, pontomedullary, and oblongata and cerebellar atrophy with clefts in the bilateral lobes (Figures 2A–H). DTI series demonstrated sparse and reduced right cerebral white matter fiber tracts compared to the left (Figures 2I–L). EEG showed a slight increase in medium-amplitude sharp waves and sharp slow complex waves in the right parietal region during sleep (Figure 3). ECG, ambulatory blood pressure monitoring, and echocardiography results were all within normal ranges.

### Molecular analysis

Pedigree analysis indicated that the proband (IV-3) is a descendant of a consanguineous marriage as his paternal grandfather (II-1) and maternal grandmother (II-4) were siblings, resulting in his parents being first cousins. His two elder sisters are phenotypically normal. Considering the health status of other family members, we hypothesized an autosomal recessive inheritance pattern.

**Figure 4:**
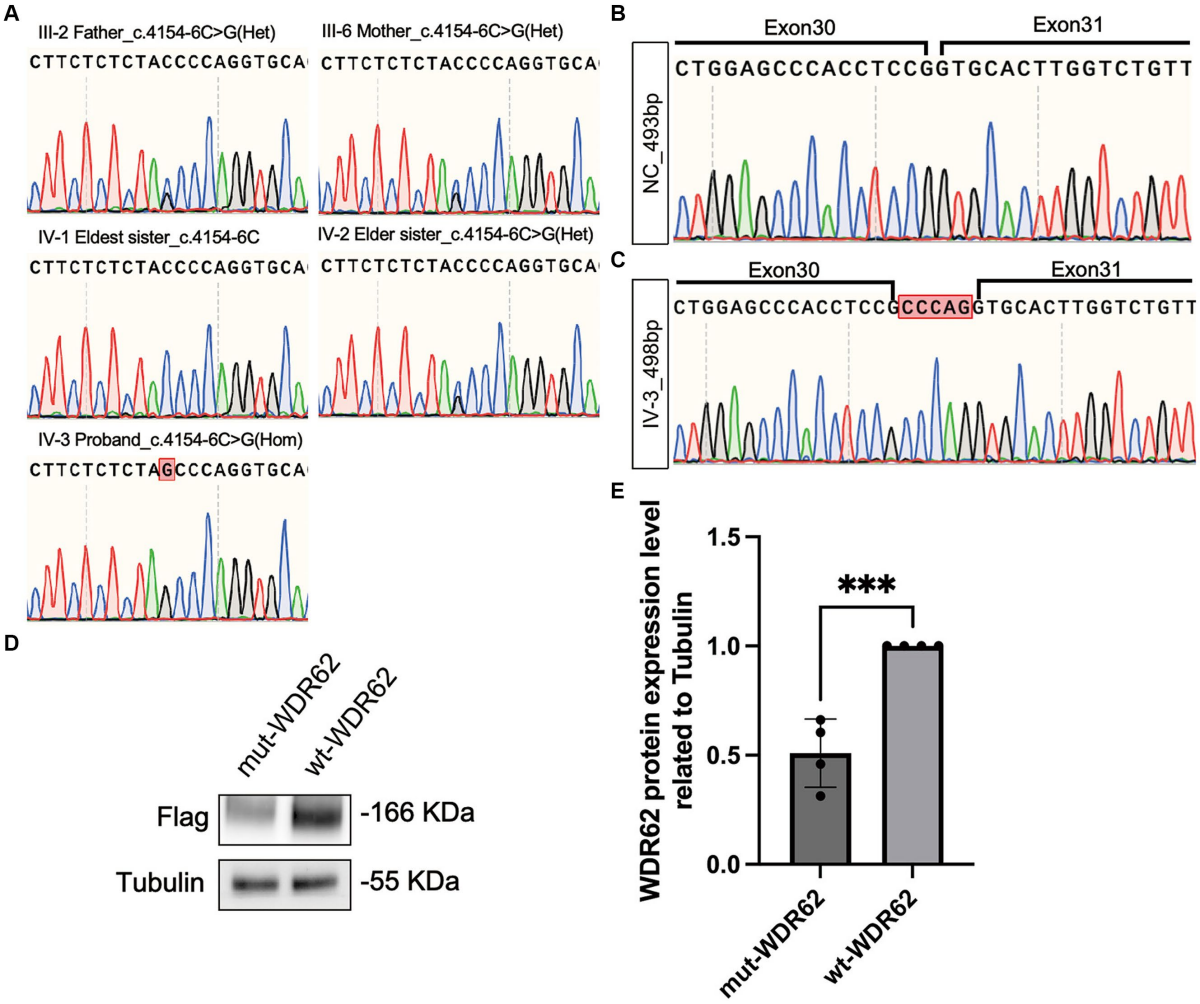
Chromatograms of *WDR62* Surrounding the c.4154–6 C > G Mutation and Specific Amplification of *WDR62* cDNA. **(A)** Chromatograms showing that the parents (III-2 and III-6) and the elder sister (IV-2) are heterozygous (Het) for the splice-site variant, the eldest sister (IV-1) is wild type for this variant, and the proband (IV-3) is homozygous (Hom) for this variant. **(B)** Chromatograms of the region flanking exons 30–31 in the negative control. **(C)** Chromatograms of the region flanking exons 30–31 in the proband, revealing a 5-bp intron retention. **(D)** An immunoblot analysis of flag-tagged WDR62 protein. **(E)** Statistical analysis between the mut-WDR62 and wt-WDR62 corresponding to **D**.

WES of the DNA of the proband revealed a novel homozygous variant c.4154-6C > G in the *WDR62* gene (NM_001083961.2). This allele frequency of the variant was exceedingly rare, recorded at 0.000007970 in the gnomAD database.^2^ Splice AI analysis^3^ indicated a high delta score of 0.96 for acceptor gain. This mutation was neither listed in ClinVar^4^ or HGMD^5^ nor reported in other major databases such as Exome Variant Server, 1,000 Genomes, or dbSNP. Sanger sequencing confirmed segregation of this mutation within the family, showing that the parents of the proband and one elder sister were healthy carriers, while the eldest sister exhibited no mutation at this site (Figure 4A).

### Functional analysis

We used MaxEntScan (20) to predict the strength of the human 5′ splice site and evaluated the c.4154-6C > G substitution. This tool rated the mutation as moderately pathogenic with a score of 6.1637. Furthermore, the dbNSFP database confirmed its pathogenicity, showing an ADA score of 0.9992 (21, 22). To understand the impact of this splice-site mutation further, we amplified and analyzed cDNA from both a negative control and the proband. Gel electrophoresis displayed a single band of approximately 493 base pairs (bp) in each sample. Nonetheless, Sanger sequencing revealed that the c.4154-6C > G mutation led to an abnormal 498-bp transcript due to the retention of 5 bp (c.4154–1 to c.4154–5 CCCAG), which was not present in the negative control (Figures 4B,C). This abnormal transcript included a premature termination codon, adding evidence to its pathogenic nature. When comparing the mutation p.Gly1385Alafs*32-truncated protein (mut-WDR62) with the wild-type (wt) WDR62 protein, we observed a significant reduction in the stability of the mutant protein (*p* < 0.0008,***). This reduction likely results from the unstable degradation of the truncated protein post-mutation (Figures 4D,E).

## Discussion

In our study, we describe a patient with MCPH2, born to consanguineous parents, who exhibited classic symptoms of MCPH2 such as microcephaly, intellectual disability, speech impairment, and epilepsy. Interestingly, the patient also presented with short stature and a limb length discrepancy, leading to a distinctive gait. These features, not typically associated with MCPH literature, hint at a possible, previously unexplored connection between cerebral malformations and limb anomalies in MCPH2, which merits further study.

Due to the wide variety of primary microcephaly subtypes (2, 23, 24), we utilized WES to streamline the diagnostic procedure. The inheritance pattern indicated an autosomal recessive trait. Our analysis uncovered a novel splice-site variant c.4154-6C > G in the *WDR62* gene, which is scarce in the gnomAD database and has not been documented in ClinVar or HGMD before. Splice-site mutations play a crucial role in genetic diseases by modifying gene expression and the diversity of the proteome. Specifically, the mutation c.4154–6 C > G located at the exon–intron junction resulted in a 5-bp intron retention, creating an abnormal mRNA transcript. We anticipated the production of an unstable and prone to degradation truncated protein (p.Gly1385Alafs*32) due to the premature stop codon. Our *in vitro* experiments confirmed a significant decrease in mut-WDR62 protein expression compared to wt-WDR62 (*p* < 0.0008), likely due to nonsense-mediated mRNA decay or reduced stability of the truncated protein.

WDR62 is primarily expressed in the forebrain, especially in the ventricular and subventricular zones (25). The asymmetrical development of the patient’s brain cortex and ventricles, as observed in MRI findings from our and other studies, remains unexplained (11, 17, 18). In our patient, the more severely dysplastic right cerebral hemisphere corresponded with a shorter left limb, suggesting that cerebral asymmetry and cortical malformations might lead to physical disabilities, such as limb length discrepancies and gait abnormalities, even though MCPH is not typically linked with malformations outside the central nervous system.

At present, there is no specific treatment for MCPH2 or care primarily focused on managing epilepsy symptoms. However, the identification of various mutations in *WDR62* splice sites associated with microcephaly or intellectual disability in the HGMD database suggests new therapeutic possibilities (12, 17, 26). Antisense oligonucleotides (AONs), which can regulate alternative splicing, have shown promise, as demonstrated by the successful treatment of spinal muscular atrophy with nusinersen (27, 28). Designing AONs to specifically target and block the novel splice sites introduced by the c.4154-6C > G mutation could help correct the abnormal splicing of pre-mRNA caused by this mutation. Furthermore, the development of CRISPR/Cas technologies (29–34), including base and prime editing, offers hopeful prospects for addressing neurodevelopmental disorders such as MCPH.

To conclude, our report on a consanguineous Chinese family with MCPH2, characterized by severe microcephaly, intellectual disability, epilepsy, and unilateral cerebral dysplasia accompanied by limb deformities, broadens the phenotypic and mutational spectrum associated with *WDR62*. This case enhances our understanding of molecular pathogenesis of MCPH2 and introduces novel approaches for its treatment.

## Data availability statement

The datasets presented in this article are not readily available because of ethical and privacy restrictions. Requests to access the datasets should be directed to the corresponding author.

## Ethics statement

The studies involving humans were approved by the Ethics Committee for Medical Research of the First Affiliated Hospital of Fujian Medical University (FYYY2006-01-1901). The studies were conducted in accordance with the local legislation and institutional requirements. Written informed consent to participate in this study was provided by the participants. Written informed consent was obtained from the individuals for the publication of any potentially identifiable images or data included in this article.

## Author contributions

HC: Data curation, Formal analysis, Investigation, Methodology, Resources, Supervision, Validation, Visualization, Writing – original draft, Writing – review & editing, Funding acquisition. YZ: Data curation, Formal Analysis, Investigation, Resources, Writing – original draft, Writing – review & editing. HW: Data curation, Formal analysis, Investigation, Resources, Writing – original draft, Writing – review & editing. NC: Data curation, Formal analysis, Investigation, Resources, Writing – original draft, Writing – review & editing. GX: Data curation, Formal analysis, Investigation, Resources, Writing – original draft, Writing – review & editing. YL: Data curation, Formal analysis, Investigation, Resources, Writing – original draft, Writing – review & editing. J-JL: Data curation, Funding acquisition, Investigation, Project administration, Supervision, Writing – review & editing, Writing – original draft.

## Footnotes

^1^ http://asia.ensembl.org/index.html

^2^ https://gnomad.broadinstitute.org

^3^ https://spliceailookup.broadinstitute.org

^4^ https://www.ncbi.nlm.nih.gov/clinvar/

^5^ https://www.hgmd.cf.ac.uk/ac/index.php
